# PEDV infection in neonatal piglets through the nasal cavity is mediated by subepithelial CD3^+^ T cells

**DOI:** 10.1186/s13567-020-00883-w

**Published:** 2021-02-17

**Authors:** Chen Yuan, Yuxin Jin, Yuchen Li, En Zhang, Penghao Zhang, Qian Yang

**Affiliations:** MOE Joint International Research Laboratory of Animal Health and Food Safety, College of Veterinary Medicine, Nanjing Agricultural University, Weigang 1, Nanjing, Jiangsu 210095 China

**Keywords:** PEDV, CD3^+^ T cells, nasal cavity, neonatal piglets

## Abstract

Porcine epidemic diarrhea virus (PEDV) primarily infects neonatal piglets causing catastrophic effects on the global pig farming industry. PEDV infects piglets through the nasal cavity, a process in which dendritic cells (DCs) play an important role. However, neonatal piglets have fewer nasal DCs. This study found that subepithelial CD3^+^ T cells mediated PEDV invasion through the nasal cavity in neonatal piglets. PEDV could replicate in the nasal epithelial cells (NECs) isolated from the nasal cavity of neonatal piglets. Infection of NECs with PEDV could induce antiviral and inflammatory cytokines at the late stage. The infected NECs mediated transfer of virus to CD3^+^ T cells distributed in the subepithelial of the nasal cavity via cell-to-cell contact. The infected CD3^+^ T cells could migrate to the intestine via blood circulation, causing intestinal infection in neonatal piglets. Thus, the findings of this study indicate the importance of CD3^+^T cells in the dissemination of PEDV from the nasal cavity to the intestinal mucosa in neonatal piglets.

## Introduction

The respiratory tract is the primary pathway for various respiratory pathogens, such as those causing influenza and pneumonia [1, 2]. As a gateway to the respiratory tract, the nasal cavity plays a vital role as the first line of defense against the invasion of such microorganisms [3]. In addition, increasing evidence for the air transmission of gastrointestinal pathogens, including Norwalk viruses and rotavirus, has been reported [4, 5]. The interactions between respiratory viruses and host nasal epithelial cells (NECs) pattern recognition receptors, may induce the release of cytokines/chemokines and stimulate an antiviral response [6]. However, little is known about the characteristics of gastrointestinal viral infection in NECs.

Porcine epidemic diarrhea virus (PEDV) is the causative agent of porcine epidemic diarrhea (PED), an acute and highly contagious enteric viral disease [7, 8]. Although PEDV can occur in swine of any age, neonatal piglets are the most severely affected [9, 10]. Symptoms in infected piglets include watery diarrhea, dehydration, and vomiting. The fecal–oral route is believed to be the primary mode of PEDV transmission [11]. Recent studies established an alternative pathway of enteric PEDV dissemination from the nasal cavity to the intestinal mucosa in swine [12]. However, the interactions between enteric PEDV and host NECs have not been elucidated. In addition, dendritic cells (DCs) located beneath the nasal mucosa of piglets can capture PEDV by forming trans-epithelial dendrites during PEDV intranasal infection [12]. Thus, DCs harboring PEDV may be vehicles for the dissemination of the virus in PEDV infection. However, neonatal piglets have fewer nasal DCs compared to swine of any older age [13]. Thus, other immune cells may participate in PEDV infection in neonatal piglets through nasal spray.

The nasal cavity of piglets is categorized into three parts: the regio vestibularis (I, II), regio respiratoria (III, IV), and regio olfactoria (V). Lymphoid tissue is randomly located in the nasal cavity. Lymphocytes have been detected in the subepithelium of the nasal cavity [14]. Moreover, it has been reported that T lymphocytes could carry PEDV [12]. The motility of lymphocytes can be altered during viral infection [15]. PEDV causes severe enteric disease in neonatal piglets, as well as milder disease in older weaned pigs. In early life, innate immune responses are not sufficiently competent to clear most pathogens or to prevent the dissemination of infections [16]. Therefore, we hypothesized that T lymphocytes may participate in PEDV infection after PEDV intranasal inoculation in neonatal piglets.

The results of the present study support an alternative pathogenic pathway of PEDV, which results in typical diarrhea symptoms in neonatal pigs. In this pathway, CD3^+^ T cells mediate PEDV infection through the nasal cavity in neonatal piglets. Our results revealed the mechanism of intranasal inoculation of PEDV in neonatal piglets, which can describe the development of strategies that are effective in controlling PEDV epidemics.

## Materials and methods

### Reagents and cell lines

Anti-pig PE-SWC3a was purchased from Abcam. Anti-pig FITC-MHCII was purchased from Bio-Rad. Dylight 488-, 594-, -conjugated secondary antibodies were purchased from MultiSciences (Lianke) Biotech Co., Ltd. Anti-pig APC-CD3ε was purchased from BD Biosciences. Human placenta Type IV was purchased from Sigma. The anti-PEDV N protein mAb was purchased from Medgene labs. Anti-pig epithelial cell marker PE- Keratin 18 (CK18) mAb was purchased from Novus Biologicals. Anti-APC (130-097-143) MiniMACS Starting kits were purchased from Miltenyi Biotec. Vero E6 cells (ATCC CCL81) were kindly provided by the Veterinary Medicine Research Center of the Da Bei Nong Group. The cell line was regularly tested for mycoplasma contamination.

### Animals

Conventional Duroc × (Landrace × Yorkshire) neonatal piglets (1-day-old) were obtained from a swine herd at the Jiangsu Academy of Agricultural Science. The neonatal piglets were born via natural farrow and fed synthetic milk. The swine herd was seronegative for antibodies against PEDV, porcine reproductive and respiratory syndrome (PRRSV), transmissible gastroenteritis virus (TGEV) and porcine circovirus type 2 (PCV2). Each experimental group of neonatal piglets was housed in a separate room with constant humidity and temperature and a 12 h light/dark cycle. All procedures and experiments performed on animals were approved by the Institutional Animal Care and Use Committee of Nanjing Agricultural University and followed the National Institutes of Health guidelines for animal experiments’ performance.

### Virus

The wild-type PEDV strain Zhejiang08 was preserved in our laboratory. The virus clustered with the emerging virulent strain [12].

### Isolation of NECs and in vitro infection with PEDV

Isolated NECs were cultured as previously described [12]. To isolate NECs from the nasal mucosa of neonatal piglets (1-day-old), the mucosa was cut into 1–2 cm pieces. The pieces were washed with Hank's balanced salt solution (HBSS) at least five times and digested in a solution containing minimal essential medium supplemented with 50 μg/mL gentamicin, 1.25 μg/mL amphotericin, 1.4 mg/mL pronase, 100 μg/mL DNase and 1% penicillin–streptomycin at 4 °C for 21 h. Cell were obtained by centrifugation. Cell suspensions were transferred to a T25 tissue culture flask and incubated in DMEM containing 4% fetal bovine serum at 37 °C for 3 h to allow attachment of fibroblasts. The cell suspension was centrifuged at 120 × *g* for 6 min and the resultant pellet was resuspended in complete bronchial epithelial growth media. The acquired NECs were seeded (1 × 10^5^) in wells of Transwell devices coated with human placenta Type IV collagen (6 µg/mL). The medium was replaced every second day. When 80% confluency was reached, the NECs were washed in PBS and then infected with PEDV at a multiplicity of infection (MOI) of 0.1. The purity of the NECs was determined by fluorescence-activated cell sorting (FACS). At 1 h post-infection (hpi), virus-containing medium was removed, the cells were washed twice with PBS, and fresh medium was added to each well. The NECs were collected at different timepoints for subsequent cytokine/Toll-like receptor (TLR) and viral analyses.

### Generation of CD3^+^ T cells

The nasal mucosa was cut into 0.5 cm pieces. The pieces were incubated in 20 mL of 10 mM EDTA in HBSS for 20 min at 4 °C. The samples were then centrifuged, discarded, and placed in digestion solution containing 4% fetal bovine serum, 2 mg/mL each of collagenase D and DNase I, and 100 U/mL dispase and slowly rotated at 37 °C for 20 min. CD3^+^ cells were obtained by density gradient centrifugation, sorted by anti-allophycocyanin (APC) microbeads, and activated by phytohemagglutinin and interleukin-2 (IL-2) for 3 d for subsequent experiments.

### PEDV infection and transmission

NECs were infected with PEDV (MOI = 0.1) at 37 °C. At 1 hpi, the cells were washed extensively to remove the unbound virus. The PEDV infected NECs were co-cultured with CD3^+^ T cells isolated from nasal mucosa at 37 °C. At 4 hpi, the number of CD3^+^ T cells harboring PEDV was determined by flow cytometry.

### PEDV intranasal inoculation

Neonatal piglets (1-day-old) of similar weight were randomly allocated into two groups (negative control [I] and PEDV infected [II] groups; n = 6 per group). The groups were housed in two separate rooms in a high-security isolation facility. Neonatal piglets in group II were challenged with 1 mL PEDV (10^6^ plaque forming units per mL) by nasal inoculation. The nasal spray device used for nasal inoculation is commonly used for vaccine absorption by the nasal mucosa and effectively atomizes particles. In group I, the same volume of PBS was inoculated in the same manner. The animals were fed with synthetic milk every 3 h throughout the experiment to meet or exceed the National Research Council requirements for nutrients and energy for this size of piglets. After challenge, neonatal piglets were observed daily for symptoms of diarrhea. Nasal cavity tissue of neonatal piglets (n = 3 for each group) was sampled and processed for immunohistochemistry (IHC) analysis 12 h after the nasal challenge. At the conclusion of the experiment, the piglets were euthanized by intravenous injection of pentobarbital sodium (100 mg/kg). Jejunum tissues were collected for IHC and immunofluorescence (IF) viral analyses.

### Quantitative reverse transcription-PCR (RT-qPCR)

Total RNA from NECs was purified using RNAiso Plus kit (TaKaRa Bio, Dalian, China) following the manufacturer's instructions. Fresh RNA (1 μg) was used as a template to synthesize first-strand cDNA with commercial oligo dT primers using Prime Script™ II 1st strand cDNA Synthesis Kit (TaKaRa Bio). PCR was performed using a SYBR Green qPCR Kit (TaKaRa Bio) in an Applied Biosystems 7500 Fast Real-Time PCR System (Life Technologies). Specific primers are shown in Table 1. Gene expression was normalized to amplify glyceraldehyde 3-phosphate dehydrogenase (GAPDH). The data were analyzed using the 2^−ΔΔCT^ method.

**Table 1:** Primers used for real-time PCR

Gene	Primer sequence (5′-3′)	Orientation
IFN-α	CTCTTCCTCCAGAAACCTGCAA	Forward
	GAGGAAGAATGGGCTTGTTAGTC	Reverse
IFN-β	CCACCACAGCTCTTTCCATGA	Forward
	TGAGGAGTCCCAGGCAACT	Reverse
IL-1β	AGAGGGACATGGAGAAGCGA	Forward
	GCCCTCTGGGTATGGCTTT	Reverse
TNF-a	GCCCTTCCACCAACGTTTTC	Forward
	TCCCAGGTAGATGGGTTCGT	Reverse
IL-6	CCTCGGCAAAATCTCTGCAA	Forward
	TGAAACTCCACAAGACCGGT	Reverse
IL-8	CCTCATTCCTGTGCTGGTCA	Forward
	TGCAAGTTGAGGCAAGAAGAC	Reverse
IL-10	CGGCCCAGTGAAGAGTTTCT	Forward
	TGCCTTCGGCATTACGTCTT	Reverse
TLRl	AGATTTCGTGCCACCCTATG	Forward
	CCTGGGGGATAAACAATGTG	Reverse
TLR2	GAGTCTGCCACAACTCAAAGA	Forward
	CAGAACTGACAACATGGGTAGAA	Reverse
TLR3	GAGCAGGAGTTTGCCTTGTC	Forward
	GGAGGTCATCGGGTATTTGA	Reverse
TLR4	TCATCCAGGAAGGTTTCCAC	Forward
	TGTCCTCCCACTCCAGGTAG	Reverse
TLR5	GGTCCCTGCCTCAGTATCAA	Forward
	TGTTGAGAAACCAGCTGACG	Reverse
TLR6	TCAAGCATTTGGACCTCTCA	Forward
	TTCCAAATCCAGAAGGATGC	Reverse
TLR7	TCTGCCCTGTGATGTCAGTC	Forward
	GCTGGTTTCCATCCAGGTAA	Reverse
TLR8	CTGGGATGCTTGGTTCATCT	Forward
	CATGAGGTTGTCGATGATGG	Reverse
TLR9	AGGGAGACCTCTATCTCCGC	Forward
	AAGTCCAGGGTTTCCAGCTT	Reverse
TLRl0	GCCCAAGGATAGGCGTAAAT	Forward
	CTCGAGACCCTTCATTCAGC	Reverse
β-actin	TGCTGTCCCTGTATGCCTCT	Forward
	CTTTGATGTCACGCACGATTT	Reverse

### Western blot analysis

Total protein from different tissues or NECs was obtained following lysis using lysis buffer. Proteins in the lysates were separated by 10% sodium dodecyl sulfate–polyacrylamide gel electrophoresis (SDS-PAGE) and transferred to a polyvinylidene difluoride (PVDF) membrane (Bio-Rad, CA). After blocking with 5% nonfat milk in Tris-buffered saline containing 0.05% Tween-20 (TBST), the membrane was incubated with the particular primary antibody, followed by horseradish peroxidase-conjugated secondary antibodies in blocking reagent. After extensive washing with TBST, immune reactive bands were analyzed by film exposure after enhanced chemiluminescence reaction (Millipore, Bedford, MA, USA).

### Flow cytometry

CD3^+^ T cells were acquired from the blood of neonatal piglets or after co-cultivation with NECs. The surface of each cell was stained with the indicated antibody. The cells were resuspended in fixation/permeabilization solution (BD Cytofix/Cytoperm kit; BD Pharmingen) and stained with PEDV N protein antibody to detect intracellular PEDV. After three washes with PBS, the cells were phenotypically analyzed by FACS.

### IHC and IFA assays

After fixation, histological sections of five blocks of nasal cavity tissue were selected according to the fractions 1/20, 1/4, 2/5, 3/5, and 4/5. Five cross-sections (I, II, III, IV, and V) of each fraction were subsequently examined by IHC to assess the distribution of PEDV using primary antibody directed against PEDV N protein. For IF staining of PEDV, tissue sections were permeabilized in 0.4% Triton X-100 in PBS for 5 min. After treatment with 5% bovine serum albumin in PBS for 1 h, PEDV located in the nasal or jejunal mucosa was immunolabeled with PEDV N protein overnight at 4 °C, followed by Alexa Fluor 488-conjugated goat anti-mouse antibody. Colonization of PEDV in the jejunum was detected by IFA with PEDV polyclonal antibody.

### Statistical analyses

Results are expressed as mean ± SD and analyzed using SPSS 17.0. One-way analysis of variance (ANOVA) was employed to determine significant differences among multiple groups. The t-test was employed to determine the differences between the two groups. Significance was expressed at a *P*-value < 0.05 or < 0.01. Data were combined from at least three independent experiments, unless otherwise stated.

## Results

### Replication of PEDV in NECs

**Figure 1:**
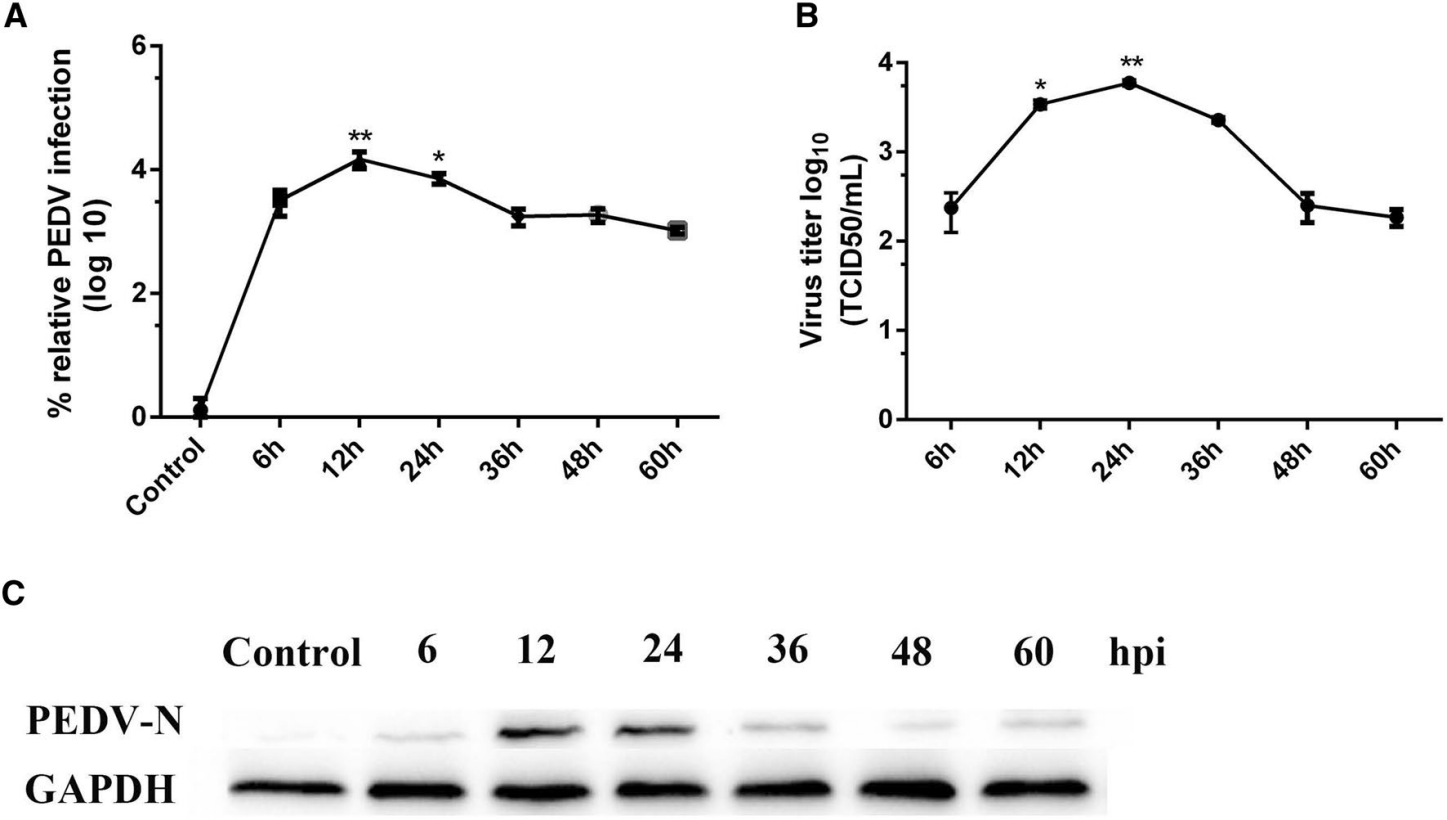
**Replication of PEDV in NECs.** **A** RNA expression levels of PEDV infected NECs at different time points was evaluated by RT-qPCR. **B** Culture supernatants of PEDV infected NECs were collected at different time points. The viral titer in the supernatant was determined with Vero cells with the results expressed as TCID_50_/mL. **C** Protein expression of PEDV in the NECs at different time points by western blotting with a mouse mAb against N protein. *Represents a significant difference relative to the 6 h group (*P* < 0.05), and **Represents an extremely significant difference relative to the 6 h group (*P* < 0.01).

NECs were successfully isolated from the nasal cavity of neonatal piglets as demonstrated by morphological examination of cultured cells. The cilia structure in some of the isolated nasal epithelium was preserved (Additional file 1A). The cytokeratin 18 epithelial cell marker was used to identify the purity of the isolated NECs. FACS analysis indicated that at least 94.6% of the cell population comprised cytokeratin-positive epithelial cells (Additional file 1B). To detect whether PEDV replicated in NECs, the cells were inoculated with PEDV (MOI = 0.1). At 6, 12, 24, 36, 48, and 60 hpi, NECs and supernatants were collected for RT-qPCR and western blot analyses, and determination of median tissue culture infectious dose (TCID_50_) to determine intracellular and extracellular virus titers. RT-qPCR showed that PEDV gene copies reached a peak of 4.12 log_10_ within 12 hpi and declined at 24 hpi (Figure 1A). TCID_50_ results revealed that viral gene replication occurred prior to virus release. The virus titer peaked at 24 hpi and then decreased gradually (Figure 1B). Western blot analysis results were similar to the TCID_50_ results. PEDV N protein was increased at 12 and 24 hpi, and then decreased gradually (Figure 1C). The collective findings indicated the replication of PEDV in NECs.

### PEDV infection induces antiviral and cytokines expression in NECs

**Figure 2:**
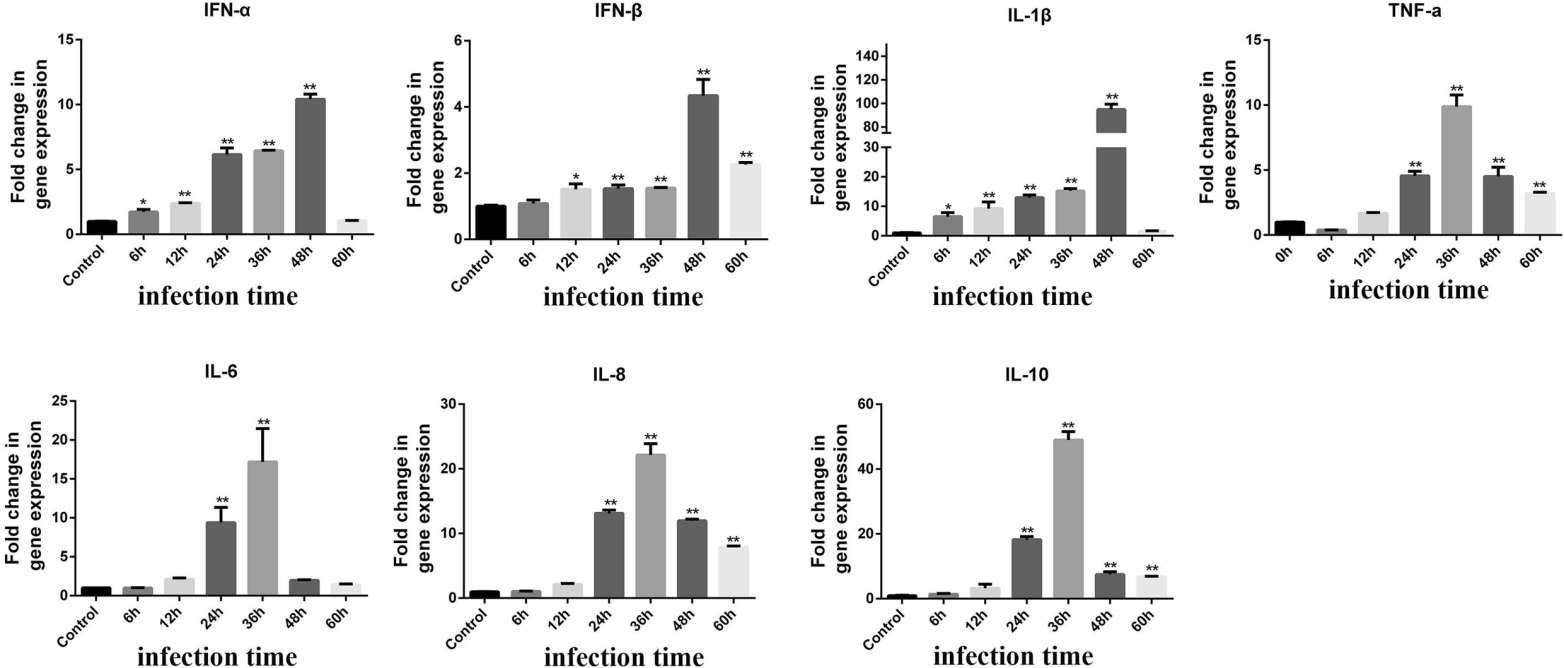
**Changes in the level of IFN-α, IFN-β, IL-1β, TNF-α, IL-6, IL-8 and IL-10 gene expression after PEDV infection of NECs for different time.** *Represents a significant difference relative to the control group (*P *< 0.05), and **Represents an extremely significant difference relative to the control group (*P *< 0.01).

Cytokine mRNA expression levels were detected by RT-qPCR and normalized to the expression of the GAPDH gene at different timepoints. As shown in Figure 2, compared with the control group, the PEDV infected group had significantly increased mRNA expression levels of interferon (IFN)-α, IFN-β, (IL)-1β, IL-6, IL-8, IL-10, and tumor necrosis factor (TNF)-α. The mRNA expression levels of TNF-α, IL-6, IL-8, and IL-10 were significantly upregulated at 36 hpi. The mRNA expression levels of IFN-α, IFN-β, and IL-1β were significantly upregulated at 48 hpi.

### Influence of PEDV infection on expression of TLR1-10 genes in NECs

**Figure 3:**
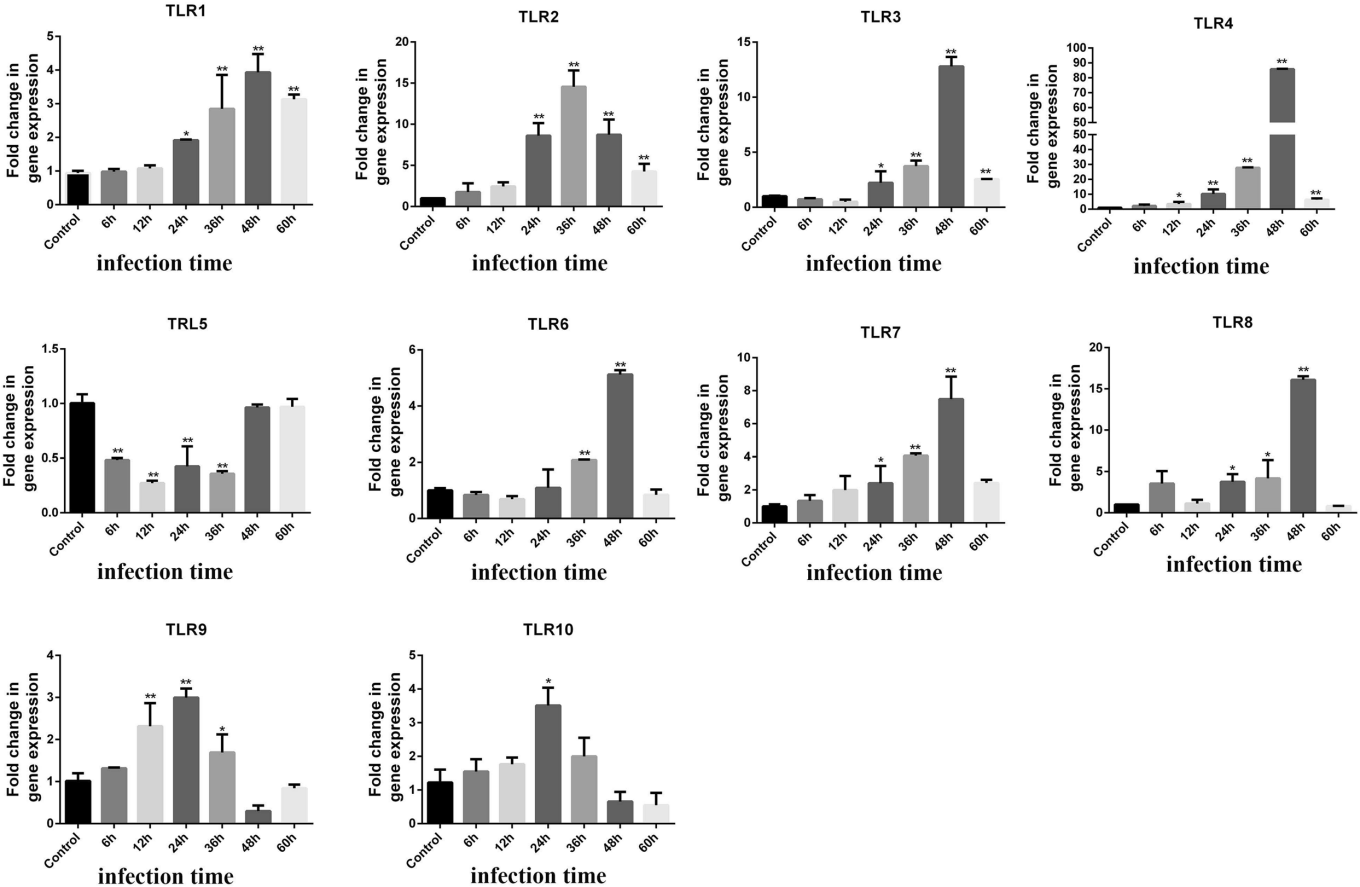
**Expression levels of TLRs after PEDV infection of NECs for different time.** *Represents a significant difference relative to the control group (*P* < 0.05), and **Represents an extremely significant difference relative to the control group (*P* < 0.01).

The mRNA expression levels of the genes encoding TLR1-10 in PEDV infected NECs were determined by RT-PCR at various timepoints following infection. As shown in Figure 3, the expression of the TLR1, TLR2, TLR3, TLR4, TLR6, TLR7, and TLR8 genes at 24, 36, 48, and 60 hpi were significantly higher than those in the control group, and their expression levels were significantly higher at 48 hpi than those at 24, 36, and 60 hpi, except for TLR2. The expression of the TLR5 gene at 6, 12, 24, and 36 hpi was significantly lower than those in the control group. Expression levels of the TLR9 and TLR10 genes were significantly higher at 24 hpi compared to those in the control group.

### Distribution of PEDV in nasal cavity after intranasal inoculation in neonatal piglets

**Figure 4:**
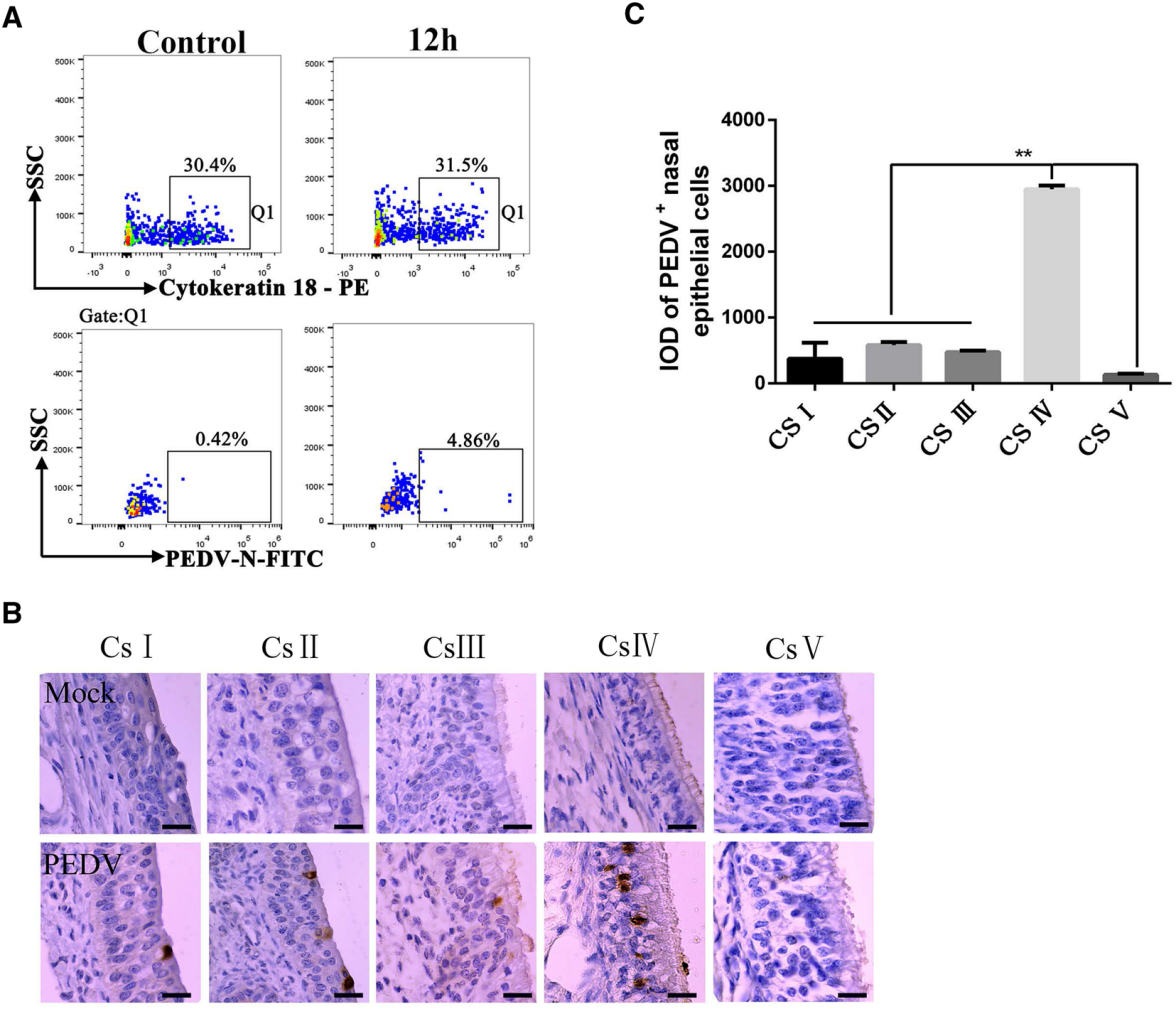
**The distribution of PEDV in nasal cavity after intranasal inoculation in neonatal piglets. A** For FACS analyses, neonatal piglets were nasally administered PEDV at indicated times. Then, individual cells isolated from the nasal mucosa were gated based on cytokeratin 18, and viral infection was detected by PEDV N protein staining, n = 3 from 3 piglets per group. **B** IHC results showed PEDV distribution in five cross-sections (I, II, III, IV and V) of the nasal cavity at 12 h post intranasal infection. The scale bar represents 20 μm. **C** Quantitative analysis of PEDV positive cells in the nasal cavity**.** All data shown are the mean results ± SD from three independent experiments. Statistical significance was tested using one-way ANOVA. **P < 0.01.

To detect the distribution of PEDV in the nasal cavity of neonatal piglets, the NECs were isolated via staining with antibody specific cytokeratin 18 after intranasal inoculation for 12 h and subjected to FACS. FACS analyses revealed that 4.86% of the NECs were positive for cytokeratin 18 (Figure 4A). IHC of nasal cavity tissue acquired at 12 hpi revealed an uneven distribution of virus-positive cells in regions I–IV of the nasal cavity, with the most virus-positive cells observed in region IV (Figures 4B, C).

### PEDV infection of CD3^+^ T cells distributed in the subepithelium of the nasal cavity in neonatal piglets

**Figure 5:**
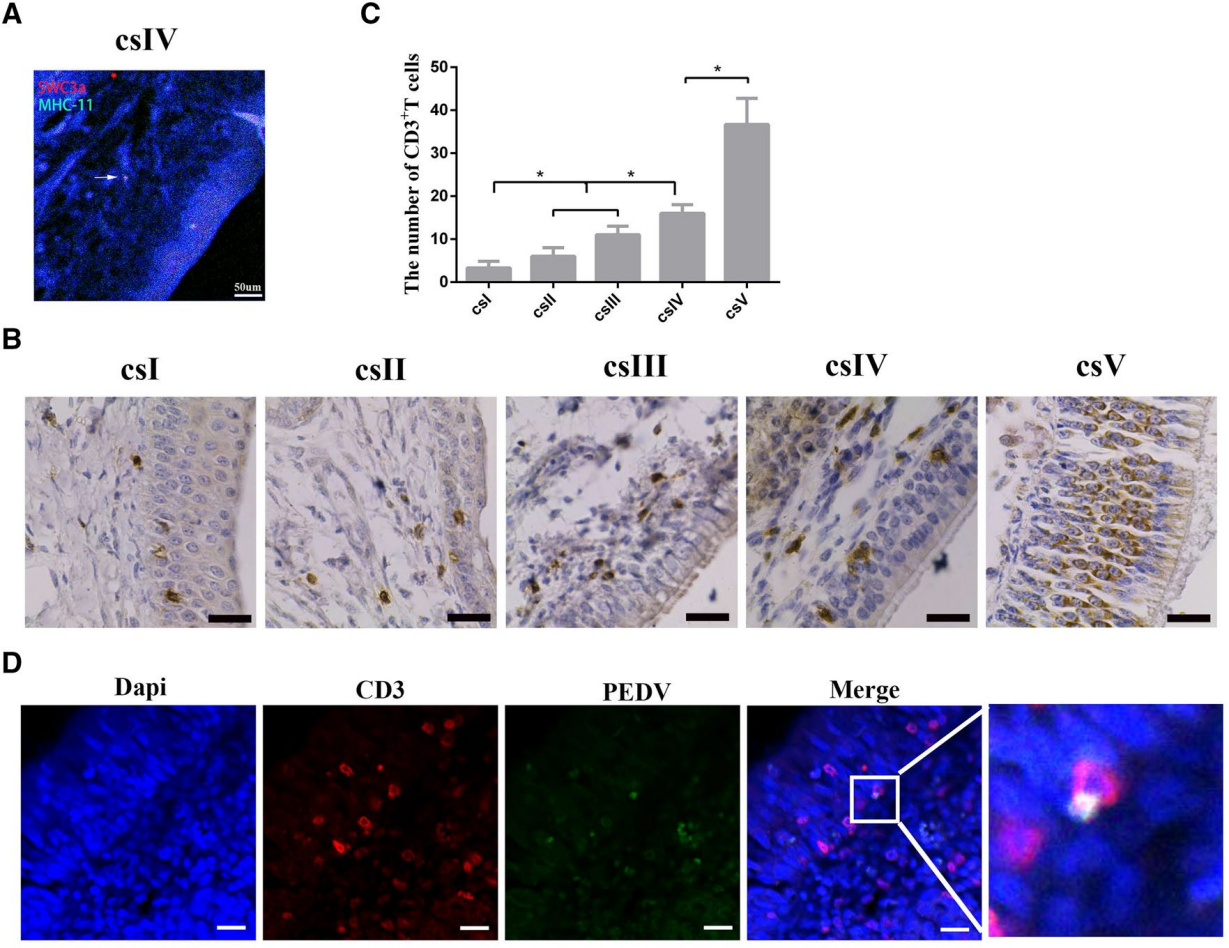
**The distribution pattern of DCs and CD3**^**+**^ **T cells in the nasal cavity of neonatal piglets**. **A** IF analysis of MHCII^+^SWC3a^+^ DCs in the nasal cavity from neonatal piglets. The scale bar represents 50 μm. **B** CD3^+^ T cells were detected in the nasal cavity from neonatal piglets using IHC. The positive cells were stained brown. The scale bar represents 20 μm. **C** Quantitative analysis of CD3^+^ T cells in the nasal cavity. The number of CD3^+^T cells was counted from five cross-sections in a unit area (40 ×) *P < 0.05. **D** IF analysis examined PEDV in CD3^+^ T cells of the nasal cavity from neonatal piglets through intranasal inoculation. The scale bar represents 20 μm. Blue, DAPI; green, PEDV; Red, CD3.

Submucosal DCs in the nasal cavity can capture PEDV and transfer the virus to T cells in piglets [12]. However, submucosal DCs and in the soft palate tonsil, nasopharyngeal tonsil and lingual tonsil are sparse in neonatal piglets [13]. We examined by IF the distribution patterns of submucosal DCs in regions IV of the nasal cavity from neonatal piglets using dual staining with antibodies specific to DCs markers. The double-positive Swc3a ^+^ MHC II^+ ^DCs stained yellow or orange. There were few DCs in the nasal cavity (Figure 5A). The finding indicates that PEDV could infect neonatal piglets via other immune cells by intranasal inoculation. IHC was used to examine the distribution patterns of CD3^+^ T cells in the nasal cavity. CD3^+^ T cells were distributed in the subepithelium of the nasal cavity from neonatal piglets, with increasing numbers of cells from the proximal to distal side of the nasal cavity (Figure 5B, C). CD3^+^ T cells derived from the nasal cavity of neonatal piglets through intranasal inoculation could contain PEDV, as visualized by double IF staining of CD3^+^ T cell and PEDV proteins (Figure 5D). These results demonstrated that CD3^+^ T cells present in the subepithelium of the nasal cavity from neonatal piglets were infected by PEDV.

### CD3^+^ T cells acquire PEDV from NECs and migrate to the intestine of neonatal piglets via blood circulation

**Figure6:**
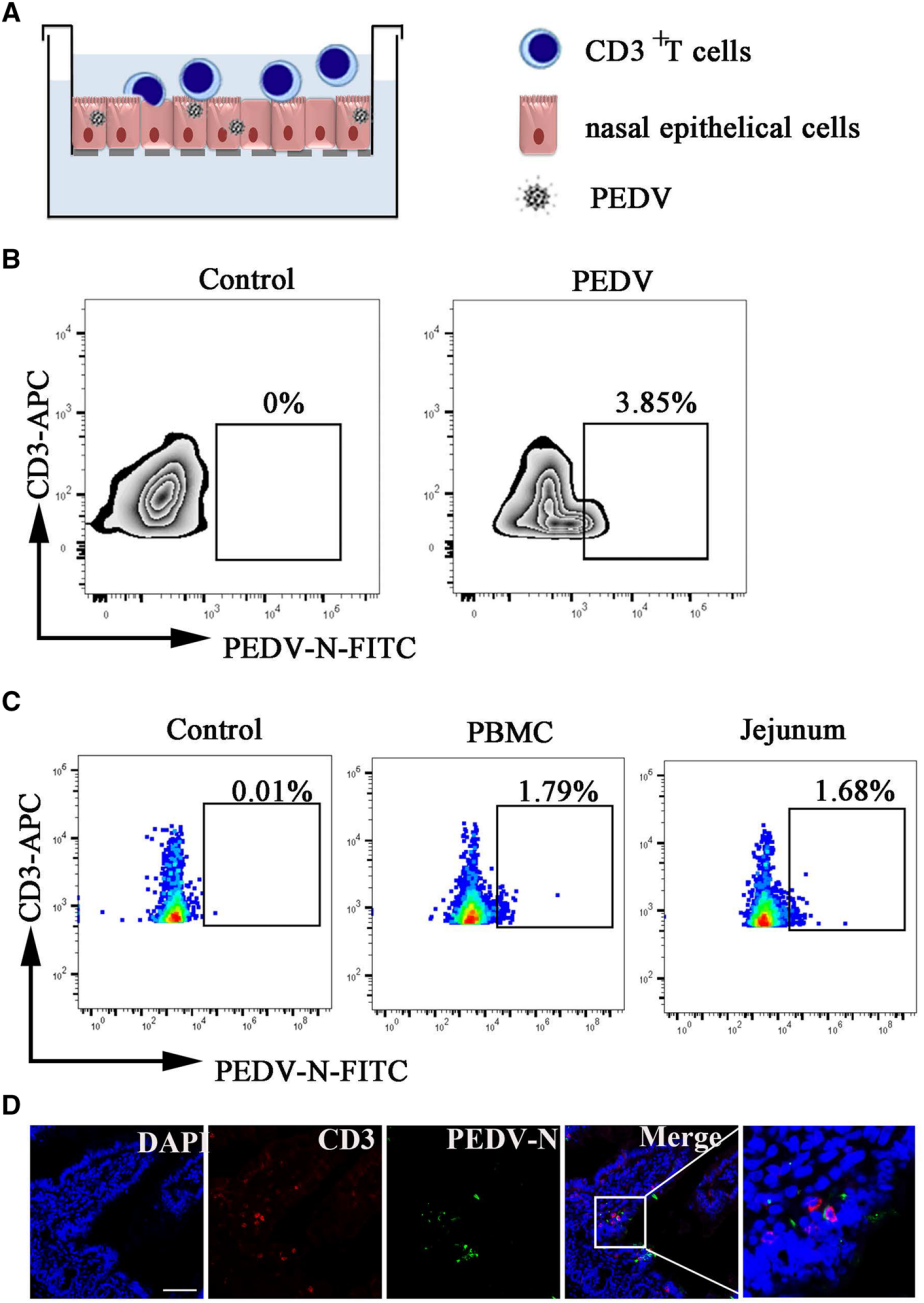
**CD3**^**+**^** T cells acquired PEDV from NECs and migrated to the intestine of neonatal piglets via blood circulation.** **A** Schematic model of PEDV-carrying NECs transmission of the virus to T cells. T cells were co-cultured with NECs by contact. **B** PEDV-infected NECs were co-cultured with CD3^+^ T cells for 4 h. Viral infection in CD3^+^ T cells was detected by PEDV N protein staining. **C** FACS analyzed the number of PEDV-loaded CD3^+^T cells derived from PBMC and the jejunum in neonatal piglets at 12hpi after intranasal inoculation. **D **IF analysis examined CD3^+^ T cells carrying PEDV of jejunum in neonatal piglets through intranasal inoculation. The scale bar represents 50 μm. Blue, DAPI; green, PEDV; Red, CD3.

To detect whether PEDV-harboring NECs allow the virus to be transferred to CD3^+^ T cells beneath the NECs, a co-culture system between the nasal epithelium and CD3^+^ T cells was established in vitro (Figure 6A). NECs infected with PEDV were co-incubated with CD3^+^ T cells sorted by anti-APC microbeads. PEDV was detected in CD3^+^ T cells after 4 h of co-culture (Figure 6B). FACS analysis revealed that the percentage of PEDV-positive CD3^+^ T cells was 1.79% and 1.68% at 12 hpi in peripheral blood mononuclear cells (PBMC) and jejunum of neonatal piglets after PEDV intranasal inoculation, respectively (Figure 6C). To confirm the FACS analysis results, we used confocal microscopy to visualize the subcellular locations of PEDV and CD3^+^T cells in the intestine of neonatal piglets after intranasal infection at 12 hpi. IFA revealed the presence of CD3^+^ T cells carrying PEDV (Figure 6D). These results showed that PEDV-carrying NECs allow the virus to be transferred to CD3^+^ T cells beneath the NECs in neonatal piglets. Subsequently, PEDV-carrying CD3^+^ T cells migrate to the intestine of neonatal piglets via blood circulation.

### PEDV intranasal inoculation in neonatal piglets causes typical PED symptoms

**Figure7:**
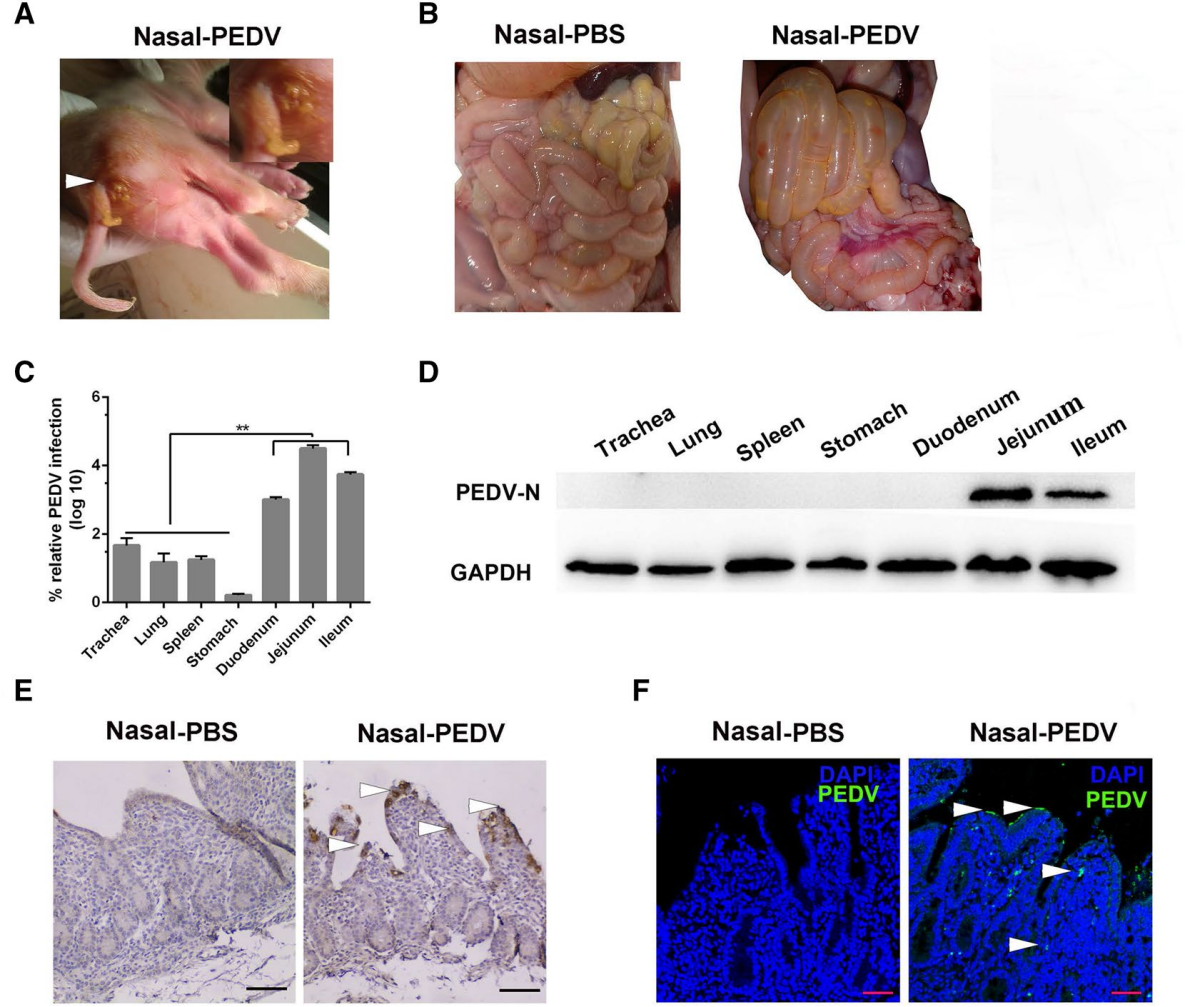
**The distribution of PEDV in neonatal piglets after intranasal inoculation.** **A** Acute watery diarrhea in neonatal piglets after PEDV intranasal inoculation at 56hpi (white arrowhead). **B** Pathological changes in the intestine after the autopsy from different groups (Nasal-PBS, Nasal-PEDV). **C** RNA expression levels of PEDV in different tissues of piglets at 60 hpi after PEDV intranasal inoculation. Data are the mean ± SD. Statistical significance was tested using one-way ANOVA. **P < 0.01. **D** Protein expression of PEDV in different tissues of diarrheic piglets a determined by western blotting with a mouse mAb against N protein. At least three independent experiments were performed. **E**, **F** IHC and IFA of the intestine of intranasally inoculated piglets at 60 h. PEDV antigen was located in the jejunum villus (white arrowhead). The scale bar represents 100 μm. Blue, DAPI; green, PEDV.

To verify that PEDV could infect neonatal piglets through intranasal inoculation, we carried out challenge experiments. The neonatal piglets were randomly divided into the control and PEDV intranasal inoculation groups (n = 3 per group). Severe watery diarrhea and vomiting were first detected in the PEDV inoculated neonatal piglets at 56 hpi. At 60 hpi, these piglets began to exhibit classical PEDV symptoms, including acute, severe watery diarrhea, depression, and lethargy. Abundant yellow, foul smelling watery stools were also observed around the perianal region of neonatal piglets (Figure 7A). The neonatal piglets were then euthanized. Pathological changes noted on autopsy of the group II animals included thinning and near-transparency of the walls of the small intestines and extended stomach filled with curdled milk (Figure 7B). Histopathological examination showed severe diffuse atrophy, fusion of villi of the small intestine, hemorrhage, and a number of inflammatory cells in group II (Additional file 2). RT-qPCR showed viral RNA expression in different tissues of neonatal piglets after PEDV intranasal inoculation. PEDV mainly colonized the jejunum and peak viral RNA titers reached 4.79 log. The viral RNA titers in the jejunum were significantly higher than those in the other tissues (Figure 7C). Western blot results further validated the PEDV level in different tissues, and a significant quantity of PEDV N protein was detected in the jejunum and ileum. However, no immune reactivity was observed with proteins of the trachea or stomach (Figure 7D). IHC and IF analyses revealed many PEDV-positive cells in the jejunum (Figures 7E, F). These results indicate that intranasal inoculation of PEDV in neonatal piglets causes PEDV intestinal infection.

## Discussion

The nasal cavity is one of the main routes of infection by pathogenic microorganisms in animals and humans [17]. Various respiratory viruses invade the nasal cavity and spread within the body. Once pathogens reach the NECs, these cells rapidly recognize the invaders and initiate local and systemic immune responses [18].The interactions between the respiratory viruses and host NECs have been reported. For example, influenza virus A infection in NECs can stimulate the expression of chemokines eotaxin [19]. NECs and bronchial epithelial cells have similar antiviral and pro-inflammatory responses during rhinovirus infection [20]. Cytokines play a vital role in protecting hosts from viral infection. However, little is known about the characteristics of gastrointestinal viral infection in NECs. The expression of the pro-inflammatory cytokines IL-1α, IL-1β, and TNF-α are significantly upregulated in Vero cells infected with PEDV [21]. PEDV infection activates nuclear factor-kappa B (NF-kB) through the TLR2, TLR3, and TLR9 pathways in porcine intestinal epithelial cells [22]. In addition, PEDV infection can induce innate immune responses in intestinal porcine jejunum epithelial cells, leading to changes in the expression of TLRs and the release of downstream cytokines [23].

In this study, the response of NECs nasal during PEDV infection was studied. The expression levels of IFN-α, IFN-β, and IL-1β in the cells at different infection times were higher than those in the control group, especially at 48 hpi. The expression levels of TNF-α, IL-6, IL-8, and IL-10 at different infection time points were higher than those in the control group, especially at 36 hpi. The effects of PEDV infection on the expression of TLR genes were also analyzed. The expression levels of most TLR family genes were significantly higher than those in the control group at different times. Contrarily, expression of TLR5 decreased with time. These results suggest the importance of cytokines and TLR gene expression in PEDV infection. These results agree with previous reports on PEDV infected epithelial cells [23]. The changes in TLR genes and the expression of cytokines in NECs infected with PEDV were preliminarily investigated to provide a theoretical reference and experimental basis for further study on the role of TLR genes in PEDV infection.

Multiple types of immune cells exist beneath the mucosal epithelium of the nasal cavity. These cells effectively prevent invasion and infection by pathogenic microorganisms [24, 25]. Paradoxically, submucosal immune cells may sometimes be harnessed by viruses to help them overcome the epithelial barrier. When this happens, a pathway is created that allows viruses to enter the submucosal layer [26–28]. A pioneering study on cytomegalovirus infection via the intranasal route defined the nasal mucosa, a natural site of viral entry, as a novel site of viral persistence [29]. Measles virus infection in the macaque upper respiratory tract is mediated by subepithelial immune cells [30, 31]. PEDV can infect piglets through the nasal cavity, where DCs play an important role [12]. Presently, numerous CD3^+^ T cells were distributed in the subepithelium of the nasal cavity from neonatal piglets. This finding implies that PEDV in the nasal cavity may be exploited by CD3^+^ T cells beneath the nasal epithelium instead of DCs.

Most viruses normally replicate in the mucosal epithelial cells at the invasion site. However, this does not result in any cellular cytopathic changes. For example, porcine alpha-herpes virus pseudorabies virus can replicate in epithelial cells of the surface mucosa and spread to the whole body via the circulatory system. Epstein–Barr virus can replicate in nasopharyngeal epithelial cells and spread to the whole body via virus-carrying B cells [32–35]. Although the replication of these viruses in mucosal epithelial cells does not produce cytopathic effects, they can serve as a source of infection with the potential to spread at any time [36, 37]. Similar to other studies, we found that PEDV could replicate in NECs and become a source of infection. PEDV-carrying NECs allow the virus to be transferred to CD3^+^ T cells beneath the NECs in neonatal piglets via cell-to-cell contact. The cell-to-cell transfer of PEDV could enable the virus to evade antibody neutralization.

Collectively, our results show that PEDV can cause typical PED symptoms in neonatal piglets through intranasal inoculation. PEDV can slowly replicate inside the nasal epithelium. This infection can induce an innate immune response in NECs, leading to changes in the expression of TLRs and cytokines. The viruses tend to be localized at the rear end of the nasal cavity. PEDV infected NECs allow the virus to be transferred to CD3^+^ T cells via cell-to-cell contact. Our results reveal the mechanism of intranasal inoculation of PEDV in neonatal piglets, which can provide more data on the development of effective strategies for controlling PEDV epidemics.

## Supplementary Information

**Additional file 1. The identification of NECs.**

**Additional file 2. Hematoxylin-eosin staining of the small intestine of the neonatal piglets.**

## Data Availability

The datasets used and analyzed during the current study are available from the corresponding author on reasonable request.

## Abbreviations

PEDV: porcine epidemic diarrhea virus
PED: porcine epidemic diarrhea
DCs: dendritic cells
PRRs: pattern recognition receptors
NECs: nasal epithelial cells
MOI: multiplicity of infection
FACS: fluorescence-activated cell sorting
TLR: toll-like receptor
CK18: cytokeratin18
PVDF: polyvinylidene difluoride
hpi: hour postinfection
TGEV: transmissible gastroenteritis virus
PRRS: porcine Reproductive and Respiratory Syndrome
APC: anti-allophycocyanin
IHC: immunohistochemistry
IF: immunofluorescence
GAPDH: glyceraldehyde 3-phosphate dehydrogenase
RT-qPCR: quantitative reverse transcription-PCR
TCID: tissue culture infectious dose
IFN: interferon
IL: interleukin
TNF: tumor necrosis factor
NF-kB: nuclear factor-kappa B
SDS-PAGE: sodium dodecyl sulfate–polyacrylamide gel electrophoresis
