# Supplementary figures and images for: PEDV infection in neonatal piglets through the nasal cavity is mediated by subepithelial CD3+ T cells
Source: Vet Res. 2021 Feb 17;52:26. doi: 10.1186/s13567-020-00883-w (PMC7888150; doi:10.1186/s13567-020-00883-w)

**a**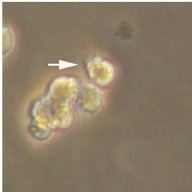**b**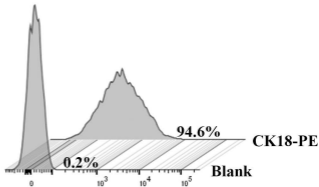

Supplement: Supplementary file 1 — Additional file 1. The identification of NECs. [file 13567_2020_883_MOESM1_ESM.pdf]

Duodenum

Jejunum

Ileum

nasal-PBS

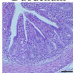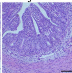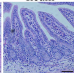

nasal-PEDV

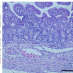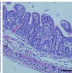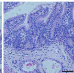

Supplement: Supplementary file 2 — Additional file 2. Hematoxylin-eosin staining of the small intestine of the neonatal piglets. [file 13567_2020_883_MOESM2_ESM.pdf]
